# Androgen Receptor Signaling and Metabolic and Cellular Plasticity During Progression to Castration Resistant Prostate Cancer

**DOI:** 10.3389/fonc.2020.580617

**Published:** 2020-10-09

**Authors:** Takuma Uo, Cynthia C. Sprenger, Stephen R. Plymate

**Affiliations:** ^1^ Department of Medicine, University of Washington, Seattle, WA, United States; ^2^ Geriatrics Research Education and Clinical Center, VA Puget Sound Health Care System, Seattle, WA, United States

**Keywords:** androgen receptor, castration-resistant prostate cancer, metabolic reprogramming ^18^F-FDG, neuroendocrine, aerobic glycolysis, fatty acid metabolism, mitochondria

## Abstract

Metabolic reprogramming is associated with re/activation and antagonism of androgen receptor (AR) signaling that drives prostate cancer (PCa) progression to castration resistance, respectively. In particular, AR signaling influences the fates of citrate that uniquely characterizes normal and malignant prostatic metabolism (i.e., mitochondrial export and extracellular secretion in normal prostate, mitochondrial retention and oxidation to support oxidative phenotype of primary PCa, and extra-mitochondrial interconversion into acetyl-CoA for fatty acid synthesis and epigenetics in the advanced PCa). The emergence of castration-resistant PCa (CRPC) involves reactivation of AR signaling, which is then further targeted by androgen synthesis inhibitors (abiraterone) and AR-ligand inhibitors (enzalutamide, apalutamide, and daroglutamide). However, based on AR dependency, two distinct metabolic and cellular adaptations contribute to development of resistance to these agents and progression to aggressive and lethal disease, with the tumor ultimately becoming highly glycolytic and with imaging by a tracer of tumor energetics, ^18^F-fluorodoxyglucose (^18^F-FDG). Another major resistance mechanism involves a lineage alteration into AR-indifferent carcinoma such a neuroendocrine which is diagnostically characterized by robust ^18^F-FDG uptake and loss of AR signaling. PCa is also characterized by metabolic alterations such as fatty acid and polyamine metabolism depending on AR signaling. In some cases, AR targeting induces rather than suppresses these alterations in cellular metabolism and energetics, which can be explored as therapeutic targets in lethal CRPC.

## Introduction

Normal cells gain distinctive capabilities to overcome the restrictions in the tissue of origin to initiate primary tumor formation (1–3). The phenotypic traits in the original environment often determine the molecular processes that drive the progression to advanced and metastatic tumors (4, 5). This is true for reprogramming of cellular and energy metabolism during cancer progression (6–8).

“The Warburg effect (aerobic glycolysis)” observed by Otto Warburg nearly a century ago is the phenomenon that cancer cells preferentially convert most glucose to lactate even in the presence of oxygen by which mitochondrial oxidative phosphorylation can proceed to generate ATP more efficiently(9, 10). His original hypothesis also emphasized that dysfunction of mitochondria is the initiating factor for cancer formation (11, 12). While not maximizing ATP production/glucose, aerobic glycolysis permits cancer cells to efficiently convert glucose into the biomass (e.g., nucleotides, amino acids, and lipids) for cell growth and proliferation (13, 14). As opposed to Warburg’s notion, most-if not all-cancer cells rely on functional mitochondria for their survival (13, 15).

**Figure 1:**
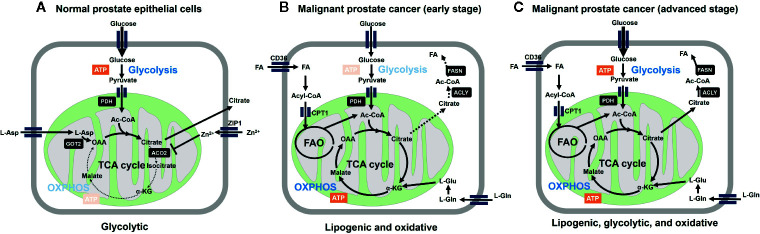
Metabolic reprogramming is involved in malignant transformation of prostatic cells. **(A)** Normal prostate epithelial cells express zinc transporter ZIP1 facilitating intracellular accumulation of zinc ion, which contributes to inhibition of m-aconitase (ACO2) at mitochondria. This inhibition results in truncation of tricarboxylic acid cycle (TCA) cycle and release of citrate to the extracellular space. Citrate production is supported by increasing the substrate pools for citrate synthase, acetyl-CoA (Ac-CoA) and oxaloacetic acid (OAA) at the mitochondria. OAA is supplied as the result of action of mitochondrial aspartate aminotransferase (GOT2) on L-aspartate. The level of mitochondrial acetyl-CoA is associated with increased expression of pyruvate dehydrogenase E1 component subunit alpha (PDHE1α) of pyruvate dehydrogenase complex (PDH). From bioenergetic point of view, normal prostatic cell is supported by aerobic glycolysis. **(B)** Marked decrease in zinc levels due to depletion of ZIP1 represents an essential early event in the development of PCa malignancy, which relieves m-aconitase to establish a complete TCA cycle. These metabolic alterations are functionally related to low citrate level and the general low avidity of ^18^F-FDG in primary PCa. Fatty acids (FA) are incorporated through CD36, followed by CPT1-mediated entry into mitochondria to serve as the substrate for fatty acid oxidation (FAO). L-Glutamine also serves as the precursor of TCA cycle intermediates after conversion into L-glutamate. ATP-citrate lyase (ACLY) cleaves citrate to produce acetyl-CoA to serve as the substrate for fatty acid synthase (FASN). **(C)** Further malignant transformation promotes glycolysis (through increased expression of glycolytic enzymes). While lipogenic trait is enhanced, multiple combinations of/all energy source pathways are theoretically available at this stage. Therefore, it is important to determine which metabolic pathway dominates for survival of given tumors for the future metabolism-based precision therapy.

Prostate cancer (PCa) is unique from a metabolic perspective. Ironically, the normal prostatic epithelial cell is one of the best cell types that fit to the original Warburg’s theory: mitochondria must be dysfunctional to get higher rate of glycolysis. Instead, primary PCa does not exhibit the Warburg effect. Contrary to other cancer cells, malignant transformation involves the conversion from energy-inefficient (“glycolytic”) secretory epithelial cells to energy-efficient (“oxidative”) PCa cells (16–20) (**Figure 1**).

Androgen receptor (AR) plays pivotal roles in both normal and malignant prostate cells. Indeed, AR transcriptional program supports PCa viability during the course from primary tumor formation to progression to metastasis. AR has the capabilities of regulating virtually all aspects of cellular metabolism (glucose, lipid, amino acid, nucleotides, etc.) (20–24). Conversely, pre-receptor control of “androgen” metabolism, which is dictated by tissue localization and abundance of steroidogenic enzymes and metabolism, ultimately determines activity of the holoreceptor for the transcriptional output (25, 26). Nevertheless, PCa exhibits specified metabolic and energetic phenotypes depending on the stage of disease progression (18, 19, 23). For example, while AR signaling persists, the transition from oxidative to glycolytic metabolism occurs during the progression to advanced PCa (27–29). Lipogenesis is continuously maintained by AR during the development of PCa (30–32). AR antagonism is highly effective in counteracting AR signaling thus altering associated metabolic programs, but tumors evolve by acquiring androgen-independent AR activation in adenocarcinoma or bypassing AR requirement through transdifferentiation to more aggressive and lethal AR-indifferent carcinomas (33). This cellular transformation results in drastic metabolic adaptation to promote aerobic glycolysis (29, 34, 35).

Understanding of the relationship between these distinctive metabolic features and AR signaling in PCa will lead to identification of metabolic vulnerabilities that offer the opportunity for diagnosis and therapy. In this review, we will characterize metabolic phenotypes of PCa in relation to AR signaling and review the current knowledge of metabolism-based imaging tools and therapeutic interventions to target cancer metabolism.

## Androgen Action in Prostate Function and Metabolism: Zinc, Truncated Tricarboxylic Acid Cycle Cycle, Citrate Metabolism

Androgens are hormones required for development and maintenance of the male reproductive system. The functions of prostatic cells in both normal and malignant condition have been characterized by the relationship to the status and availability of androgen and its cognate receptor AR. Upon binding to androgen, AR which is otherwise sequestered in the cytoplasm translocates to nucleus and acts as sequence-specific dimerized transcription factors (36).

The unique metabolic processes in the prostate are well adapted to fulfill the major function as a secretory tissue to generate prostatic fluid comprised of high concentration of citrate along with zinc, lipids, and kallikrein enzymes including prostate-specific antigen (PSA) (37, 38) (**Figure 1**). Typically, citrate is either retained and oxidized in the mitochondria to generate energy as an essential intermediate in the citric acid cycle, or is exported into the cytoplasm where it is cleaved by ATP citrate lyase (ACLY) to generate acetyl-CoA, which is used for fatty acid (FA) synthesis (39).

The normal human prostate retains the capability of accumulating the highest levels of zinc in any soft tissue of the human body through expression of specific zinc transporters (ZIP1–4 for uptake and ZnT1–10 for release) (40). High levels of mitochondrial zinc inhibit mitochondrial aconitase, resulting in truncation of tricarboxylic acid cycle (TCA) cycle at the first step of citrate oxidation (17, 41). Androgen signaling enhances citrate production by increasing the substrate pools for citrate synthase, acetyl-CoA and oxaloacetic acid (OAA) at the mitochondria. The level of mitochondrial acetyl-CoA is associated with increased expression of pyruvate dehydrogenase E1 component subunit alpha (PDHE1α) (42). Aspartate uptake is through the excitatory amino acid transporter SLC1A1/EAAC1 (43). Followed by transamination processes to generate OAA at the mitochondria (44). Mammalian cells typically produce ~38 ATP/glucose through the combined actions of glycolysis and TCA cycle oxidation on glucose. On the other hand, the normal prostatic epithelia can generate only ~14 ATP/glucose due to truncation in TCA cycle resulting in the loss of ~24 ATP/glucose (19).

Marked decrease in zinc levels due to depletion of ZIP1 represents an essential early event in the development of PCa malignancy (45), which relieves mitochondrial aconitase to establish a complete TCA cycle (18, 19). These metabolic alterations are functionally related to low citrate level and the general low avidity of ^18^F-FDG in primary PCa (46, 47).

## AR Drives PCa by Regulating Central Metabolism

Multi-omics studies (transcriptome, proteomics, cistrome, and metabolome) define the AR as a master regulator that orchestrates cellular metabolism to fuel proliferation and growth of PCa cells (20–22, 48, 49). Specifically, AR transcriptionally regulates multiple pathways of energy and biomass supply, including glycolysis, mitochondrial respiration, metabolism of FA (synthesis, ß-oxidation, and uptake), nucleotides, amino acids, and polyamines. Thus, drastic metabolic alterations are expected to inevitably follow AR inhibition and re/activation during the progression to lethal CRPC along with AR antagonism therapy.

### Glucose Metabolism

AR determines bioenergetic traits through regulation of components in glycolytic pathway (GLUT1, HK1, HK2, and PFK2/PFKFB) and pyruvate flux into mitochondria (PDH, MPC2) (21, 42, 50). AR signaling increases expression of glucose-6-phosphate dehydrogenase (G6PD) which directs glucose-6-phosphate from glycolysis to the pentose phosphate pathway (PPP) for generation of NADPH and nucleotide precursors (51). The conversion from pyruvate to lactate is catalyzed by LDH proteins including AR-target LDHA (52, 53). Hyperpolarized ^13^C magnetic resonance spectroscopic imaging (MRSI) demonstrates that *in vivo* conversion [1-^13^C] lactate into [1-^13^C] pyruvate occurred more efficiently in PDX models of AR-driven CRPC than those of AR-negative PCa (54). Monocarboxylate transporter MCT4 is upregulated in CRPC and contributes to completion of successful aerobic glycolysis through secretion of lactate. Indeed, MCT4-targeting antisense oligonucleotides (ASO) provide significant tumor suppressive activity in cellular and xenograft models of CRPC (55). Overall, AR is capable of promoting both glycolysis and pyruvate oxidation, indicating AR’s predominant roles in both glycolytic and oxidative PCa tumors.

### FA Metabolism

AR regulates FA metabolism by controlling expression of more than 20 enzymes involved in many aspects of lipid metabolism, including uptake, trafficking, synthesis, and degradation (32, 49).

AR and the master regulator of lipid homeostasis sterol regulatory-element binding protein (SREBP) regulate each other in a positive feedback system (32, 49, 56, 57). SREBP’s transcriptional targets include ELOV6 and SCD1 (58) while fatty acid synthase (FASN) and ACC (acetyl-CoA carboxylase) are co-targeted by both SREBP and AR (49, 59). Thus, AR activation accelerates FA synthesis, particularly as the form of mono-unsaturated and saturated FA (31, 32). Conversely, AR inhibition leads to marked reduction of *de novo* lipogenesis and permits incorporation of dietary FA enriched in polyunsaturated FA which are prone to lipid peroxidation when subjected to oxidizers such as arsenic trioxide (60, 61).

In addition to citrate oxidation, fatty acid oxidation (FAO) is the dominant energy producing pathway through decomposition of *de novo* or exogenous FA (62–65). Both FA synthesis and FAO have been recently shown to play key roles in cancer cell growth and proliferation (49, 63). This is an apparently contradictory situation where catabolism and anabolism of the same group of metabolites co-exist in the same cells. Also, FA synthesis and FAO have traditionally been considered incompatible due to the inhibitory effects of malonyl-CoA (the product of ACC1 which serves as the substrate for FASN) on carnitine palmitoyltransferase 1 (CPT1) in the carnitine shuttle (the rate limiting step for the transport of FA into the mitochondria) (66). Nevertheless, pharmacological or genetic inhibition of FASN resulted in decreased FAO as well as oxygen consumption, suggesting the existence of simultaneous FA synthesis and oxidation in the cells (67). Moreover, combined inhibition of FASN and FAO produced additive therapeutic effects in PCa, demonstrating that two pathways coexisting and feeding each other in some situations (65, 68). More excitingly, CPT1A-mediated FAO is reportedly to linked to epigenetics by supplying acetyl-CoA for histone acetylation (69).

On the other hand, there is growing body of evidence that PCa utilizes exogenous FA derived from diet or adipocytes (70–72). Blockade of this incorporation by CD36 inhibition is antitumorigenic (71). These reports emphasize that pharmacological intervention in FA metabolism has therapeutic benefit.

### Amino Acids

AR regulates amino acid catabolism through expression of amino acid transporters (LAT1, LAT3, ASCT1, ASCT2) (43, 73–76). LATs and ASCTs are for bulky and small neutral amino acids, respectively. In particular, ASCT2 prefers the conditionally essential amino acid glutamine as the substrate. Glutamine undergoes glutaminolysis to generate TCA cycle intermediates *via* glutamate production as an alternative energy source, providing pharmacological glutamine starvation as a therapeutic strategy (74, 77–80).

### One Carbon Metabolism Network

AR regulates one-carbon metabolism network consisting of the two folate cycle pathways (DHFR, GNMT, SARDH), and methionine cycle (MAT, AHCY) which interact with trans-sulfuration pathway (CBS, CTH) and polyamine synthesis (ODC1, AMD1) (81–83). The methionine cycle contributes to the formation of *S*-adenosyl-methionine (SAM), the universal methyl donor for protein and DNA methyltransferase reactions(84). Thus, this metabolism may contribute to AR-driven malignant progression by promoting DNA synthesis and changing DNA and histone methylation status (81). As discussed below, availability of SAM determines neuroendocrine PCa (NEPC) status which is AR independent (85).

### Addiction to Altered Metabolism

Dependence of AR on reprogrammed metabolic characteristics occurs in FA and ornithine metabolism. AR signaling is blunted when genetic or pharmacological inhibition of the rate-limiting enzymes in the pathways, such as ODC1, FASN, and CPT1 (67, 68, 82).

## Metabolic Plasticity in Relation to Anti-AR Therapy and the Resistance Mechanisms

**Figure 2:**
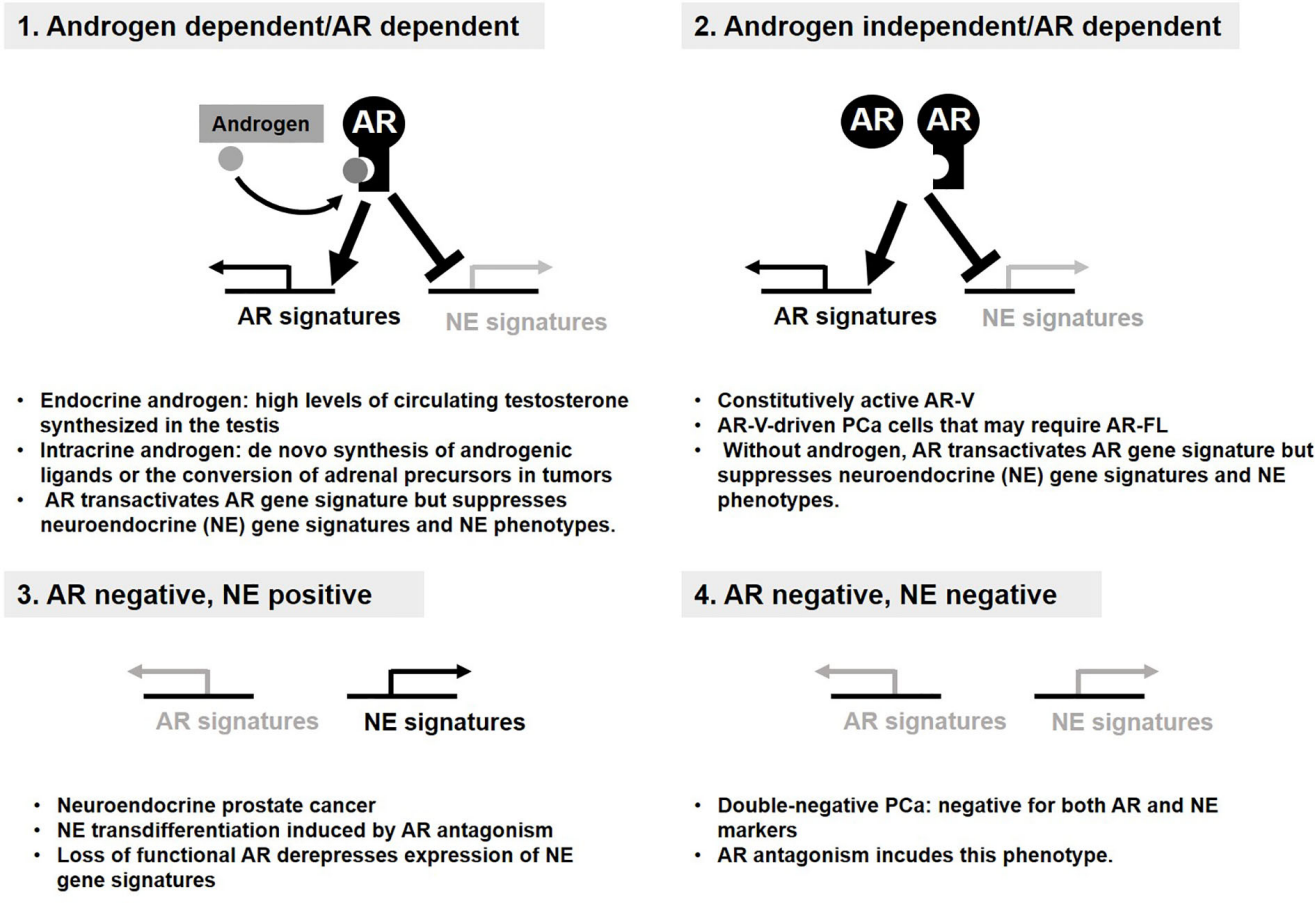
Androgen receptor (AR) status defines four distinct groups of prostate cancer (PCa). Four distinct groups of PCa display the resistance mechanism to anti-AR therapy. AR signaling supports survival and growth of PCa and suppresses transdifferentiation into neuroendocrine. (1, 2). Loss of AR signaling derepresses expression of NE gene signatures required for NE phenotypes (3). Double-negative PCa bypasses AR requirement without NE phenotype (4).

**Figure 3:**
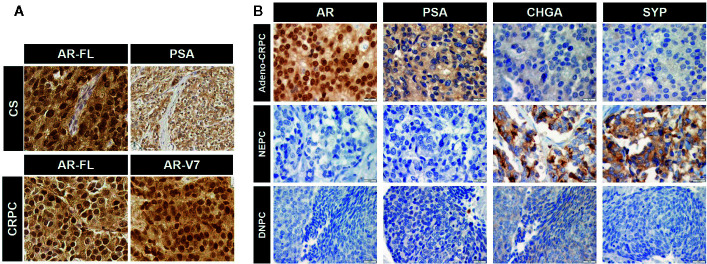
Immunohistochemical images for the expression of androgen receptor (AR), prostate-specific antigen (PSA), and neuroendocrine PCa (NEPC) markers. **(A)** Expression of full-length AR (AR-FL) and PSA in castration-sensitive (CS) PCa and AR-FL and AR-V7 in CRPC. Note uniform nuclear staining of AR-V7. CS and CRPC correspond to group (1) and (2) in **Figure 2**, respectively. **(B)** Expression of specific markers for each CRPC type. CRPC adenocarcinoma are positive for AR and its transcriptional target PSA but negative for NEPC markers chromogranin A (CHGA) and synaptophysin (SYP). NEPC is positive for NEPC markers and negative for AR and PSA. DNPC is negative for AR, PSA, and NEPC markers (image courtesy of Dr. Colm Morrissey at University of Washington). Representative images for the data in **Figure 2** (2: Adeno-CRPC, 3: NEPC, 4: DNPC). Scale bar=20 µm.

Since 1950s, inhibition of AR activity has remained a mainstay in the treatment of advanced PCa (86–89). Although most patients with PCa initially respond to AR inhibition, they eventually develop castration-resistant PCa (CRPC) (36, 90, 91). The emergence of CRPC usually involves reactivation of AR signaling(92–97), which is then further targeted by as androgen synthesis inhibitors (abiraterone) and AR-ligand inhibitors (enzalutamide, apalutamide, and daroglutamide) (98, 99). Nevertheless, resistance to these agents and progression to lethal disease are essentially universal by developing adaptive resistance to these target therapies through two distinct groups of mechanisms based on AR dependency (100) (**Figures 2**, **3**). Continued AR activation occurs by multiple mechanisms including increased AR expression in close association with enhanced intracrine or paracrine androgen synthesis (**Figure 2**, Group 1), AR gene mutations enabling promiscuous ligand interaction, and expression of constitutively active AR variants (AR-Vs)(Group 2) (100–103). AR antagonism can also promote lineage crisis and cellular plasticity to bypass AR blockade and generate neuroendocrine PCa (NEPC)(Group 1, 2→Group 3, Group 4→Group 3) (33, 104–106). Transformation into treatment-induced NEPC (t-NEPC) requires lineage plasticity in adeno-PCa to bypass AR blockade along with three major events: (i) The loss of AR expression. (ii) Alternative splicing of REST transcript by SRRM4 leading to the loss of REST activity that represses neuroendocrine gene expression. (iii) Activation of NE transcription factors e.g. ASCL1 and BRN2 that determine the commitment to a NE lineage (105–108). Among many other factors, EZH2 stands out to regulate NEPC-specific gene expression through epigenetic machinery (105). Importantly, N-MYC forms a transcription repressor complex with EZH2 to repress AR transcription program (109). It is noteworthy that AKT1-mediated phosphorylation drives a non-epigenetic mode of EZH2 action as AR coactivator to support androgen-independent AR activation during CRPC development and progression. Another emerging cell type is double negative PCa (DNPC)(Group 1, 2→Group 4), which is negative for both AR and neuroendocrine markers and may represent an intermediate phenotype between AR expressing adenocarcinoma and the neuroendocrine phenotype (110).

### Alteration in Pre-Receptor Control of Dihydroxytestosterone Metabolism

5α-Reduction of testosterone (T) in prostate results in the formation of the more potent ligand dihydroxytestosterone (DHT) to activate AR. Thus, ADT is the frontline treatment and directed toward disruption of T-DHT-AR axis by suppression of gonadal T by medical or surgical castration (26). Resistance to gonadal T depletion namely CRPC is associated with AR activity which is achieved by a gain-of-function in AR itself and/or sufficient intratumoral amounts of T and DHT to activate AR (25, 26, 101). Metastatic prostate tumor cells synthesize their own androgens through *de novo* steroidogenesis, which involves upregulation of enzymes required for stepwise synthesis from cholesterol to T and DHT (93, 111). Another strategy requires adrenal synthesis and supply of dehydroepiandrosterone (DHEA) and its sulfate (DHEA-S) which are converted to Δ^4^-androstenedione (AD) by 3β-hydroxysteroid dehydrogenase/isomerase (HSD3B1) in PCa (25, 26). AD is converted to DHT through canonical (AD→T→DHT) or alternative (“backdoor”) pathway involving the intermediate androstenedione to form DHT. Importantly, a gain-of-function mutation in HSD3B1 (N367T) leads to stabilization of the enzyme which confers two distinct survival benefits to PCa (112, 113). This variant supports CRPC status to bypass depletion of gonadal testosterone by facilitating the synthesis of AD thus flux to DHT from adrenal DHEA and DHEA-S (112). The resistance to anti-AR therapy is acquired by this variant which more efficiently converts the androgen synthesis inhibitor abiraterone into the precursor of potent AR agonist (113).

### AR Dependent Mechanisms: Full-Length AR (AR-FL) and AR-V7 Specific Signaling?

As discussed above, AR is the master regulator of cellular metabolism. The questions remain as to whether AR-Vs are simply a constitutively active substitute for liganded-AR-FL to control cellular metabolism. Do AR-V7 and AR-FL differentially contribute to a selective adaptation during metabolic rewiring that occurs in CRPC progression? To answer this question, androgen-treated LNCaP and LNCaP engineered to co-express AR-V7 were used to extract AR-FL and AR-V7 signaling, respectively (114). AR-V7 specific metabolic signatures include reduced citrate level as a result of enhanced utilization rather than a failure to synthesize citrate. AR-V7 enhanced glycolytic flux more effectively than AR-FL with enhanced conversion of glutamine to citrate *via* reductive carboxylation (114). These findings suggest that AR-V alters flux of a subset of metabolites to provide growth advantage. As of yet, no such data has been generated to address the functional contribution of endogenous AR-Vs to bioenergetic phenotypes.

### AR Indifferent CRPC: Drastic Metabolic Changes Are Associated With Cellular Lineage Alterations

MYC family proteins regulate virtually all genes involved in glycolysis not only by controlling their express levels but shifting alternative splicing toward glycolytic isoform PKM2 over PKM1 (115, 116). Moreover, MYC, increases mitochondrial export of acetyl groups as the form of citrate and the resulting acetyl-CoA contributes to histone acetylation by histone acetyltransferase GCN5 (117). Indeed, there exists the interplay between the epigenetic landscape and metabolism (118). For example, pyruvate generated from glycolysis is the main substrate for acetyl-CoA, a central metabolite coordinating the activity of the histone acetyltransferase (HAT) enzymes. Increased expression of the histone lysine demethylase KDM8 is observed in the context of treatment-induced NEPC and transactivated expression of EZH2 (119). Mechanistically, the KDM8-mediated PKM2 nuclear translocation results in the transcriptional activation of glycolytic program, including GLUT1, HK2, PKM2, LDHA, etc.) and downregulation of genes for pyruvate dehydrogenase complex (PDHA1 and PDHB1) to reduce the direction of pyruvate to mitochondria. As a proof of concept, inhibition of glycolysis lead to growth inhibition (119). Phosphoglycerate dehydrogenase (PHGDH) is the first enzyme branching from glycolysis in the serine biosynthesis which involved in one-carbon metabolism to supply S-adenosyl methionine (SAM) (120). SAM in turn serves as the substrate for DNA and protein methyltransferases. Cancer metabolism is linked to epigenetics in this scenario. Upregulation of PHGDH is common in NEPC thus facilitating methylation-related epigenetic modifiers such as EZH2 (105).

## Positron Emission Tomography–Based Metabolic Phenotyping

**Figure 4:**
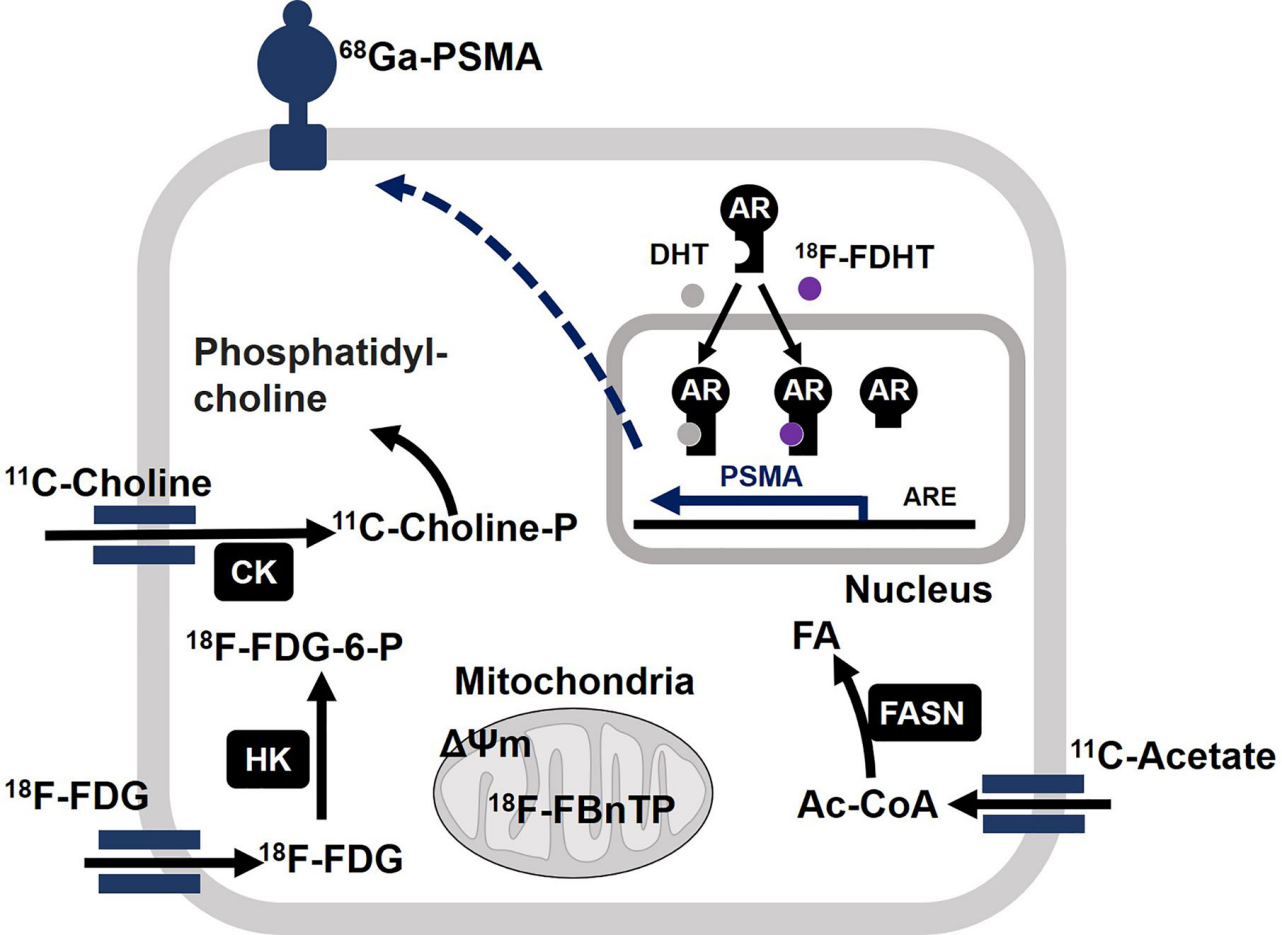
Molecular basis of actions of positron emission tomography (PET) radiotracers in prostate cancer (PCa). Acetate is converted to acetyl-CoA (Ac-CoA) which serves as a substrate for FASN to produce fatty acids (FA). After enzymatic modification by choline kinase (CK), ^11^C-choline is incorporated into cell membrane as the form of phosphatidylcholine. After incorporation into cell, ^18^F-FDG undergoes phosphorylation by hexokinase (HK) and accumulates as the form of ^18^F-FDG-6-P. Mitochondrial membrane potential (ΔΨ_m_) drives accumulation of 4-[^18^F]fluorobenzyl triphenylphosphonium (^18^F-FBnTP) at mitochondria. ^18^F signal is indicative of respiration-competent functional mitochondria. Binding of ligand dihydrotestosterone (DHT) activates full-length AR as a transcriptional factor to upregulate target genes such as PSMA. Accordingly, the presence of full-length AR can be monitored by ^18^F-FDHT. It is noteworthy that constitutively active AR variant fails to bind to ^18^F-FDHT. Accordingly, ^18^F-FDHT negativity does not necessarily mean tumors are negative for any form of AR. ^68^Ga-labeled antagonistic ligand for PSMA can be used to monitor tumors with active AR signaling.

*In vivo* metabolic phenotyping involves the steps for profiling and characterizing energetic phenotypes of tumors, which has a great diagnostic value for PCa patients. In this regard, ^18^F-FDG, ^18^F- or ^11^C-labeled acetate, and ^18^F- or ^11^C-labeled choline represent the three most studied positron emission tomography (PET) radiotracers in the PCa field (121, 122). Biochemical characteristics of tumors correlate well with uptake of each radiotracer (**Figure 4**). Acetate uptake is increased concomitantly with elevated FASN activity (123, 124). Upregulation of choline kinase (CK), which is associated with malignancy, promotes phosphorylation of choline to be incorporated in cellular membrane as the form of phosphatidylcholine (125, 126). While both acetate and choline uptake serve as a basis of powerful PET imaging, it has been well accepted that PCa displays less avidity to ^18^F-FDG (46, 47). However, largely depending on the disease phase, 84% of mCRPC patients have at least one ^18^F-FDG positive metastasis. Moreover, 85% of ^18^F-FDG positive metastasis displayed positivity for another tracer ^18^F-fluorodihydrotestosterone (^18^F-FDHT) used as indicator of AR(-FL) expression (29). On the other hand, prostate specific membrane antigen (PSMA) is “imageable” AR-target gene product (127). Thus, ^68^Ga-PSMA-PET imaging reflects relative changes in treatment-dependent AR activity thus providing high diagnostic values (128). The expression levels of glucose uptake–associated genes, including GLUTs and hexokinases to provide a genomic rationalization for the previously reported ^18^F-FDG avidity of PSMA-suppressed PC tumors such as NEPC and DNPC (35, 129). Non-invasive imaging tools have not been available for oxidative phosphorylation in tumors. Oxidative tumors can be monitored by the agent 4-[^18^F]fluorobenzyl triphenylphosphonium (^18^FBnTP) whose uptake is driven by mitochondrial membrane potential (ΔΨ_m_) (130). Thus, combined use of these diagnostic tools will be powerful to characterize bioenergetic phenotypes of PCa tumors and determine treatment options.

## Tumor Metabolism in CRPC Is Observed Across Various Cancer Types?

As discussed above, PCa develops adaptive resistance to AR-targeting therapy through two distinct groups of mechanism based on AR dependency. In addition to alterations in AR structure of function, AR-dependent mechanism involves aberrant pre-receptor metabolism of steroids which is arguably unique to CRPC. AR-independent paths include transdifferentiation into NEPC and DNPC. Nevertheless, advanced CRPC, NEPC, and DNPC are ultimately addicted to aerobic glycolysis which is associated with high avidity of FDG in PET scan (29, 35). Ironically, Warburg effects occur in virtually all types of cancers and may represent the final form of tumor metabolism (13, 23, 131). Consistently, systems biology approach was used to analyze the expression of metabolic genes across 20 different cancer types and their impact on clinical outcome, which demonstrates that downregulation of mitochondrial genes is associated with the worst clinical outcome across all cancer types (132). Interdependence of AR and FASN drives AR-dependent CRPC progression (67), but overexpression of FASN is the rule rather than the exception in many types of cancers (133). Cancer cells appear to undergo a tissue-specific metabolic rewiring, which converges toward a common metabolic landscape. One may ask “What metabolic programs differentiate one cancer type from the others?”. A recent report from the Vander Heiden group specifically addressed this issue by testing whether tissue-of-origin dictates cancer dependence on specific metabolic pathways (134). Mouse models of pancreatic ductal adenocarcinoma (PDAC) and non-small cell lung carcinoma (NSCLC) have the same genetic background with Kras mutation and TP53 deletion, While PDAC tumors have decreased branched-chain amino acids (BCAA) uptake, NSCLC tumors incorporate free BCAAs into tissue protein and use BCAAs as a nitrogen source while PDAC tumors have decreased BCAA uptake. Expression pattern of BCAA metabolizing enzymes in original tissues reflect these metabolic differences in tumors, arguing both tumor genetics and tissue context define cancer dependence on specific metabolic pathways (134). While TP53 and RB1 are commonly tumor suppressive in many cancer types (135), their combined inactivation promotes cell plasticity in PCa to undergo NEPC differentiation (136, 137). In this scenario, PCa-specific metabolic status might permit this lineage transition.

## Therapeutic Interventions

Two biological events are emerging as hallmarks of cancer: reprogramming of energy metabolism and evading immune destruction (1). The latter is an active area of research as cancer immunotherapy. Metastatic PCa with CDK12 inactivating mutations (3-7% incidence) has durable responses to PD-1 blockade by checkpoint inhibitors (138, 139). As for targeted therapy in cancer metabolism, gain of function mutations in isocitrate dehydrogenases (IDH1 and IDH2) result in the production of the “oncometabolite” 2-hydroxyglutarate (140, 141). Targeting mutant IDH is attractive but limited in PCa: IDH mutations account for only 1-2% of PCa incidence, which is much lower than other tumors, e.g. glioma (~50%) (142, 143). For PCa, dysregulated FA metabolism, which is mechanistically linked to aberrant AR and/or SREBP signaling (49, 144), has multiple candidate factors for pharmacological inhibition, including SREBP (fatostatin) (145), acetyl-CoA carboxylase (ND-646, GS-0976) (146, 147), and SCD1 (Merck Frosst Cpd 3) (148). IPI-9119 (67) and TVB-2640 (80) are selective FASN inhibitors for potential clinical use. Treatment with IPI-9119 led to disruption of the interdependence between AR and FASN and extensive reduction in AR signaling (67). Energy disruptors aim to reduce intracellular ATP level by inhibiting glycolysis or disturbing mitochondrial mechanisms leading to oxidative phosphorylation (33, 149). Several options are available for pharmacological inhibition of glucose metabolism: glucose uptake (phloretin) (150) and glycolytic enzymes (3-bromopyruvate and Koningic acid for GAPDH) (151, 152). Complex I (NADH–quinone oxidoreductase) is the largest respiratory complex of the mitochondrial oxidative phosphorylation system (153). Complex I inhibition has been shown to be a potential clinical repressor of prostate growth based on early correlative and retrospective studies in men with PCa who had received metformin for treatment of their associated diabetes mellitus (154, 155). Thus, mitochondrial energy metabolism emerges as cancer therapy target (156). In addition to direct inhibition on oxidative phosphorylation (BAY87-2243 and IACS-010759 for complex I) (157, 158), the strategies can be developed to prevent entry of the precursors of TCA cycle intermediates into mitochondria. Glutamine utilization can be prevented by inhibiting glutamine uptake and metabolism (CB839 for glutaminase and V9302 for ASCT2) (159–161). CPT1 inhibition prevents the entry of FA into mitochondria and thus downstream FAO (65). On the other hand, MSDC-0160 inhibits pyruvate entry into mitochondria by mitochondrial pyruvate carrier (144).

Therapeutic targets in cancer metabolism in many cases exist even in the normal cells, which adds potential toxicity and non-specificity to drugs targeting metabolic pathways (162). It is necessary to define their specific action in the context of tumor initiation and progression. The successful application of metabolic inhibitors will lie in accurate metabolic phenotyping and stratification of tumors to predict which respond to the given drugs.

## Discussion

We have described how PCa is unique from other cancers from the metabolic point of view. In addition, AR signaling persists in normal and malignant prostatic cells except for when AR antagonism triggers the transition to highly glycolytic AR-indifferent carcinoma. AR determines virtually all aspects of cellular metabolism while a selected phenotype is dominant depending on the stage of disease progression. Accordingly, the question remains as to what directs AR toward specified metabolic preference. The underlying mechanisms may include the presence of AR-Vs, differential actions of AR co-regulators, epigenetics, and tumor microenvironment. Understanding and targeting the selective AR-metabolome axis may provide the unique therapeutic opportunity for AR-driven CRPC which is resistant to current anti-AR therapy.

Except for targeting mutant IDHs, metabolic inhibitors are potentially active regardless of tumor genetic subtype and thus beneficial to the large majority of men with CRPC who are not currently candidates for precision medicine (e.g., DNA repair defects for PARP inhibitors or CDK12 loss for immunotherapy) (138, 163). Nevertheless, appropriate tumor imaging at spatial resolution (e.g., use of PET radiotracers) may facilitate select effective metabolic therapy by determining what bioenergetic phenotype dominates in tumors (glycolytic, lipogenic, or oxidative) (121, 122). For instance, FASN inhibition may be selected when ^11^C acetate uptake suggests tumors are lipogenic. High avidity to ^18^F-FDG is supported by expression of glycolytic gene signature in NEPC, providing a rationale to target glucose metabolism for therapy. Tumor plasticity adds another layer of complexity to PCa as it develops and spreads. Altered metabolic pathways may be dispensable or indispensable depending on the stage of tumor progression. This is true for *de novo* FA synthesis whose pharmacological inhibition is detrimental in some cases (antitumorigenic regardless of availability of exogenous lipids) but tolerable in others (e.g., rescued by lipids derived from diet and adipose tissues) (67, 70).

To develop effective metabolism-based target therapy (164), it is crucial to identify metabolic pathways that define the stage of tumor progression depending on AR and cellular lineage status. The success of future therapies may be enhanced by the combination of the prescribed metabolic inhibitors such as metformin and statins (155, 165).

## Author Contributions

TU and SRP conceived and wrote the manuscript. All authors contributed to the article and approved the submitted version.

## Funding

VA merit review award (I01BX003324) and DoD award (W81XWH-17-1-0323 W81XWH-20-1-0146, W81XWH-17-1-0484) to SRP. NCI P50 CA097186, and the Institute for Prostate Cancer Research at the University of Washington and Fred Hutchinson Cancer Research Center.

## Conflict of Interest

The authors declare that the research was conducted in the absence of any commercial or financial relationships that could be construed as a potential conflict of interest.
